# Assessing Corrosion Effects on the Electrical Performance of Wearable Photovoltaic Cells: A Comparative Analysis of Current Consistency and Resistance

**DOI:** 10.3390/ma18020267

**Published:** 2025-01-09

**Authors:** Amit Talukder, Charles Freeman, Caroline Kobia, Reuben F. V. Burch

**Affiliations:** 1 Department of Textiles, Merchandising, and Interiors, The University of Georgia, Athens, GA 30602, USA; at67309@uga.edu; 2 Department of Human Sciences, Mississippi State University, Starkville, MS 39762, USA; ck645@msstate.edu; 3 Department of Fashion Merchandising, Texas Christian University, Fort Worth, TX 76129, USA; 4 Department of Industrial and Systems Engineering, Mississippi State University, Starkville, MS 39762, USA; burch@research.msstate.edu

**Keywords:** photovoltaics, corrosion, electrical resistance, wearable textiles, durability

## Abstract

Wearable photovoltaic (PV) cells offer a sustainable and lightweight solution for energy-harvesting applications, including safety gear and protective textiles. Despite their growing adoption, the application of PV cells in marine environments is limited due to the corrosive conditions that can degrade performance. This study evaluates the impact of corrosion on commercially sourced PV cells by analyzing maximum current and electrical resistance. This study used eight samples of two types of PV panel cells and tested them in corrosion conditions, and current and electrical resistance values were recorded. A paired sample *t*-test was used to assess variations in current and electrical resistance, while a repeated MANOVA compared the performance of two sample types during corrosion. The results reveal that corrosion significantly reduced current values and increased electrical resistance in Sample Type (1), while Sample Type (2) remained relatively stable. The MANOVA findings show a significant decrease in current for both samples, though the magnitude of reduction is similar between types. However, when combining both sample types, corrosion has no significant effect on electrical resistance. These results highlight the need for developing more durable, corrosion-resistant PV cells suitable for marine applications, emphasizing their potential for sustainable and practical use in harsh environments.

## 1. Introduction

Wearable electronics encompass a series of smart electronic accessories and systems developed to be worn on the human body. The captivating attribute of wearable electronics lies in their capability to fulfill the ever-growing demand for providing assistance that augments sensing, data storing, processing, and transferring but not sacrificing comfort [1,2,3,4]. In addition, there has been substantial progress in reducing the power requirements of devices and enhancing their capabilities. This progress makes it possible to harness potential energy from diverse sustainable sources like the environment and body motion. Notably, energy harvesting technologies, such as triboelectric (uses mechanical energy from the environment), piezoelectric (uses vibration), thermoelectric (uses heat), and photovoltaic (uses sunlight) power generation, are rapidly rising fields in today’s technologies [5,6,7,8,9,10]. The pursuit of sustainable and autonomous gadgets that operate independently of traditional power sources or regular battery replacements highlights the significance of energy harvesting [11].

Meanwhile, autonomous devices have become essential in numerous industrial applications, such as navigation, communication, and fishery. Regarding navigation and location-specific fishing, commercial fishermen rely on portable GPS devices because these devices are convenient for performing these operations. On the other hand, autonomous underwater vehicles (AUVs) are self-propelled free-diving vehicles that use sound navigation and range for marine creature classification, meaning they need a reliable power supply to run their onboard equipment as they are usually powered by fuel cells or batteries [12]. Therefore, the demand for reliable and sustainable energy sources for these AUVs corresponds with the increasing emphasis on sustainability in fuel worldwide [13,14,15].

Wearable photovoltaic (PV) technology, also called solar technology, is expanding rapidly among practitioners [16]. Wearable PV cells are well-renowned and perform best with textiles due to their adaptability; they use the photovoltaic effect to convert sunlight into electrical energy [17]. These cells facilitate diverse applications, including energy-harvesting apparel, safety equipment, protective textiles, and military outfits [18]. However, several factors are necessary to consider before adopting PV cells in wearable applications because various degradation and failure phenomena influence these cells’ durability, launderability, and efficiency [19]. A crucial engineering challenge in applying these cells in marine environments happens due to the harsh external environment, which results in most companies producing wearable PV for terrestrial applications, not marine ones. Corrosion in marine conditions predominantly results from chloride ions, which markedly accelerate the electrochemical reactions responsible for corrosion. In these settings, moisture from the atmosphere or direct exposure to seawater generates an electrolyte that promotes the conduction of electric current between anodic and cathodic regions on the metal surface. This process may result in localized corrosion occurrences, such as pitting, characterized by forming small, deep pits on the metal surface, undermining its structural integrity. Elevated humidity, temperature variations, and corrosive agents, including sulfides and biological entities, can expedite corrosion, leading to heightened deterioration of materials utilized in PV cells [20]. With these corrosive environmental challenges, few PV cells are commercialized for marine applications. In addition, salt from perspiration during physical exercise can cause metallic parts of wearable electronics to corrode; this is also true for seawater in marine applications [21]. When exposed to the salt content of seawater, marine wearables may lose some of their effectiveness because of the possibility of electric shocks or total signal loss from corrosion. A few researchers conducted studies in this area, but not directly related to flexible, wearable PV cells applicable for clothing for the marine environment [20,22,23]. In 2021, Y. Zhang and Yuan conducted experiments on the performance of PV cells in a saline sea environment for the shipping industry application. As salt spray and seawater are the most influential factors, the authors analyzed and revealed influences on the electrical output values of PV cells, altering these results according to changes in concentrations. The synergistic impact of salt spray reduces electrical output to around 6% because it reduces the solar irradiance; oppositely, seawater increases by 20% [23]. Therefore, the performance of flexible wearable PV cells for textiles remains uncertain. Consequently, exploring how product developers might integrate PV wearables into textiles for the marine environment is essential.

Wearable PV cell corrosion testing standards do not yet exist, despite several competing products being available in the market. For instance, in 2021, researchers tested PV modules, which are collections of PV cells, to assess how the marine environment affected electrical outputs. Their experiment also lacks standardized performance testing procedures. Therefore, the researchers designed their experimental platform consisting of a main workbox, a sample holder, a modular lighting system, and a water circulation system (capable of salt spraying at a rate of 30 L/h). Following testing, there was a 6% decrease in electrical production [23]. In this way, several researchers developed their own method to continue their testing, and some followed the testing procedure applied to textile materials to evaluate their reaction to salty air in coastal environments [24,25]. Performing an electrical resistance test after a corrosion assessment on PV cells is crucial for assessing the effects of corrosion on electrical performance and longevity because corrosion can increase electrical resistance, reducing current flow and decreasing overall PV cell performance [26]. Another reason is that corrosion testing can result in interconnecting degradation, leading to electrical resistance and compromised PV cell performance [27,28]. Thus, manufacturers ascertain whether the wearable PV cell adheres to necessary performance standards by evaluating electrical resistance after a corrosion test. Additionally, electrical resistance measurements assist in identifying whether modifications to material composition are required to enhance the performance and dependability of these cells against corrosion-related issues. To evaluate the electrical resistance characteristics of wearable electronic textiles, researchers often depend on AATCC test methods, specifically, AATCC EP13 [29]. This study adopted this same standard to perform this test.

The purpose of this study is to compare the maximum current consistency in milliampere (mA) and electrical resistance in kilo-ohm (kΩ) of commercially sourced wearable PV cells pre- and post-corrosion testing conditions. We hypothesized that the findings would reveal how PV cells perform in marine applications. These findings would also help product developers determine PV cells’ applicability in marine products.

## 2. Materials and Methods

### 2.1. Samples

**Figure 1:**
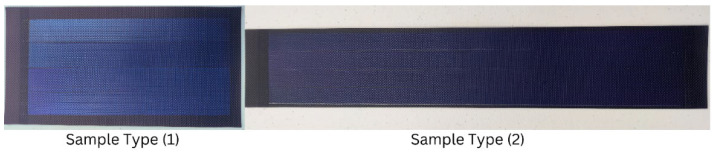
Amorphous PV cells.

This study investigates two varieties of thin-film, roll-up, bendable amorphous PV panel cells that were purchased from JIANG Solar Company in Baoding, China. The technical characteristics of the two types of PV cells are different. Sample Type (1) has a small size of 7.87″ × 3.94″, a minimum thickness of 0.04″, weighs just 28 g, has a power output of 1 watt, and requires an input voltage of 6 volts. Sample Type (2), on the other hand, is bigger, measuring 14.96″ × 2.56″, weighs slightly more (36 g), has a greater wattage (1.2 watts), an input voltage of 6 volts, and the same thickness (0.04). For comparative analysis and additional assessment, these variations in size, weight, and power are crucial (see Figure 1).

### 2.2. Testing Measurements

#### 2.2.1. Current Measurement and Electrical Resistance Test

**Figure 2:**
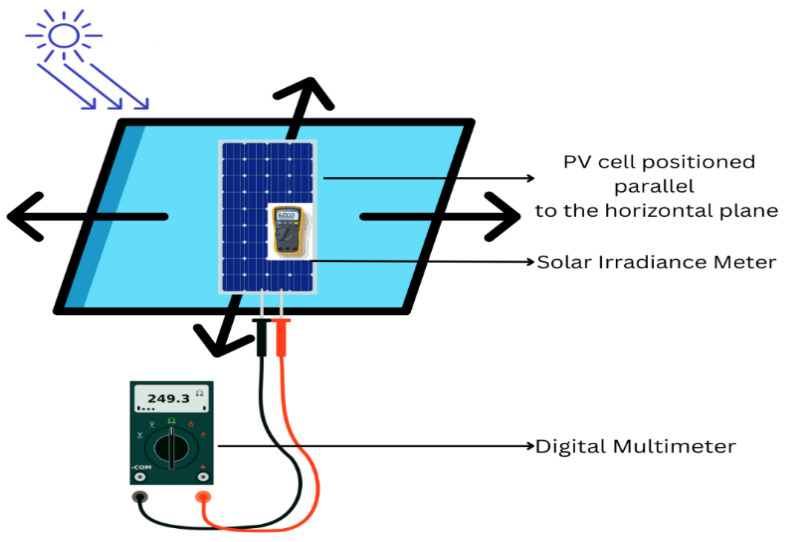
Measuring wearable PV cells’ current and electrical resistance using a digital multimeter and solar irradiance meter.

For measuring current values, the researchers used a digital multimeter (Klein Tools^®^ MM600, Lincolnshire, IL, USA) to assess the current values by attaching probes to the amorphous PV cells, adhering to the methodology of one piece of the prior literature [30]. The digital multimeter, configured to the “Current” mode, was connected with the red lead to the positive terminal and the black lead to the negative terminal. To ensure solar irradiance, a critical determinant of PV cell efficacy, they employed a Fluke^®^ IRR1 solar irradiance meter (Fluke Corporation, Everett, WA, USA), which precisely quantifies irradiance, tilt angle, and temperature [31]. These parameters are crucial for consistency in power generation for PV cells. In one previous piece of research, Veligorskyi et al. (2018) documented 950 W/m^2^ of solar irradiance at ideal conditions utilizing a photo-resistive sensor [32]; they assessed irradiance at various angles: below 30°, above 30°, and in shaded conditions at a consistent PV cell temperature of 29 °C. Our study recorded the initial measurements of PV cell temperature of 29 °C, with the cells positioned horizontally (0° angle) within a regulated enclosure (refer to Figure 2). Current measurements were repeated three times, and average values were used to verify precision and uniformity.

The researchers flatly placed the PV cells on the surface to measure electrical resistance, ensuring uniformity during the subsequent electrical resistance assessment. The digital multimeter was configured to the “Resistance” mode, with two probes linked to it. These probes were connected to both sides of the cells, with one probe corresponding to the positive terminal and the other to the negative terminal. The measurement was performed three times for each instance, and the resulting values were averaged to ensure robust and precise data. For both measurements, the readings were taken before and after corrosion testing.

#### 2.2.2. Corrosion Test

**Figure 3:**
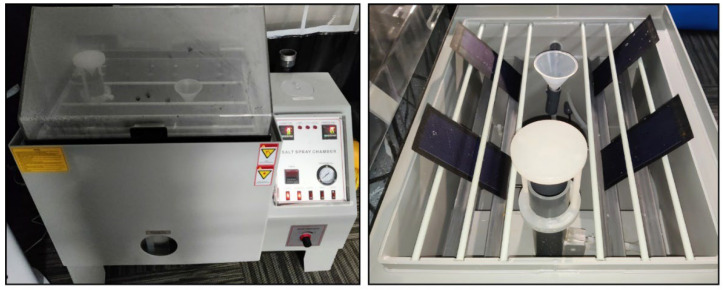
Wearable PV cells in salt spray chamber.

The researcher followed the corrosion testing technique outlined in ASTM B117-11 [24], which describes the salt spray (fog) test parameters [25]. Room temperature is kept at 25 ± 1 °C, with a relative humidity level of 50 ± 5%. Wearable PV cells were positioned in a VEVOR^®^ Salt Water Spray Chamber (Ontario, CA, USA) to assess their resilience to salt spray in a controlled corrosive environment (see Figure 3). Then, the researcher made a salt solution by combining five parts sodium chloride (NaCl) (laboratory-grade, obtained from VEVOR^®^, Ontario, CA, USA) with 95 parts distilled water (procured from Ozarka^®^, Hawkins, TX, USA), targeting a 5% concentration. The necessary mass of NaCl was determined using the formula:

0.053 × Mass of Water = Mass of NaCl required

According to this formula, 17.5 L of water and 0.9275 kg of NaCl were utilized. The pH of the solution was modified utilizing a pH-sensing electrode, a reference electrode, and a pH meter (PmoYoKo^®^), resulting in an initial pH of 7.39. To sustain the pH within a range from 6.5 to 7.2 during the experiment, many drops of 1 M laboratory-grade hydrochloric acid were introduced. Then, the researcher maintained the solution at 35 °C (95 °F), which was measured by a Thermocouple (Fluke^®^, Everett, WA, USA) and monitored pH to maintain consistency. Four specimens of Sample Type (1) were oriented at a 30° angle from the vertical, aligned with the fog flow, to guarantee optimal exposure (refer to Figure 3). Compressed air was treated in a water-filled tower at 46–49 °C (114–121 °F) to mitigate cooling effects during atomization. Upon completion of the setup, the salt spray chamber was initiated, generating a continual fog, and the samples were subjected to exposure for 24 h. This period approximates four days of exposure to maritime environments, predicated on six hours of sunlight daily. During the examination, the researcher observed parameters to confirm they stayed within the designated range, while samples were consistently assessed for corrosion signs such as rust or discoloration. Following 24 h, the samples were extracted, examined for corrosion, and documented. The technique was conducted for four samples of Sample Type (1) and (2), culminating in eight samples being tested and documented.

### 2.3. Data Analysis

SPSS Version 26.0 (IBM Corp., Armonk, NY, USA) was used to analyze current consistency and electrical resistance data. Then, the authors used a statistical analysis tool named the paired sample *t*-test, which researchers usually use to measure the performance of a sample before and after completing the program. Here, it is used to evaluate the differences before and after corrosion testing, as described in the prior literature [33]. Finally, the authors conducted a repeated measure mixed analysis of variance (MANOVA), usually a statistical tool that compares the means of multiple dependent variables across multiple groups, to assess and compare the performance of the two sample types. This analytical technique was guided by Allcoat et al. in 2021 [34].

## 3. Results

This study aims to compare the maximum current and electrical resistance of commercially sourced wearable PV cells pre- and post-corrosion testing conditions. This comparison will provide insights into the applicability of long-lasting, sustainable, and functional wearable PV cell-integrated textiles for the marine environment. As mentioned, a paired sample *t*-test and a repeated MANOVA test were conducted for data analysis.

### 3.1. Comparison Between the Current Values Pre- and Post-Corrosion Testing

In terms of current values measurement, two sample types were subjected to a paired sample *t*-test before and after corrosion at α = 0.05. Pre-corrosion current values (M = 183.01, SD = 8.11) and post-corrosion current values (M = 181.31, SD = 8.12) differed statistically significantly for sample type (1); according to this study, t(7) = 4.41, *p* = 0.003 (two-tailed) (see Table 1 and Table 2). With a 95% confidence interval, between 0.79 and 2.61 the average value decrease was 1.70. For sample type (2), on the other hand, the results showed a mean decrease of 0.64 in current values with a 95% confidence interval but no statistically significant difference between pre-corrosion current (M = 271.25, SD = 7.60) and post-corrosion current (M = 270.61, SD = 8.57), t(7) = 1.377, *p* = 0.211 (two-tailed) (see Table 1 and Table 2). So, it can be concluded that current consistency values decreased significantly after the corrosion test for sample type (1), *p* < 0.05. However, regarding sample type (2), it can be concluded that corrosion testing had no significant effect on current consistency values, *p* > 0.05.

### 3.2. Comparison Between Electrical Resistance Values Pre- and Post-Corrosion Testing

A paired sample *t*-test was performed to evaluate electrical resistance in two sample types before and after corrosion testing at each cycle with α = 0.05. Table 3 and Table 4 reveal a significant difference in pre-corrosion electrical resistance (M = 0.313, SD = 0.099) compared to post-corrosion resistance (M = 0.333, SD = 0.093), t(7) = 2.507, *p* = 0.041 (two-tailed) for sample type (1). For sample type (2), no significant differences were noted between pre-corrosion resistance (M = 0.417, SD = 0.230) and post-corrosion resistance (M = 0.430, SD = 0.224), t(7) = 0.623, *p* = 0.533 (two-tailed) (see Table 3 and Table 4). So, it can be concluded that electrical resistance values increased significantly after the corrosion test for sample type (1), *p* < 0.05. However, regarding sample type (2), it can be concluded that corrosion testing had no significant effect on electrical resistance values, *p* > 0.05.

### 3.3. Comparing Pre-Post Current Value Change During Corrosion Across Types

The MANOVA revealed no substantial interaction between the two interventions; one is corrosion phases, and another is sample types, Wilks’ Lambda = 0.82, F(5, 10) = 3.11, *p* = 0.100, and eta^2^ = 0.182. A notable main effect was detected for the corrosion phases, means before and after corrosion, Wilks’ Lambda = 0.48, F(5, 10) = 15.059, *p* = 0.002, and eta^2^ = 0.518, signifying that both sample types exhibited a reduction in current across the corrosion phases (refer to Table 5). The findings indicate that the corrosion process significantly affected current consistency, resulting in a gradual drop. Table 6 demonstrates a statistically significant main effect of corrosion, F(1, 14) = 15.059, *p* = 0.002, eta^2^ = 0.518, which indicates the variation in both independent and dependent variables. This result concludes that there is a change in current values across the corrosion phases. However, another important aspect was that there was also a non-significant interaction effect between corrosion and types, F(1, 14) = 3.11, *p* = 0.100, eta^2^ = 0.182, which means the corrosion has an impact on current values but no difference between types. Therefore, from the MANOVA analysis, no significant difference was found between all samples, which helps to conclude that current values reduced before and after corrosion. It is valid for both sample types.

### 3.4. Comparing Pre-Post Electrical Resistance Value Change During Corrosion Across Types

The MANOVA indicated no substantial interaction between the two interventions; one is corrosion phases, and another is sample types, Wilks’ Lambda = 0.998, F(5, 10) = 0.031, *p* = 0.862, and eta^2^ = 0.002. Also, there was no notable main effect for before and after corrosion, Wilks’ Lambda = 0.927, F(5, 10) = 1.103, *p* = 0.311, and eta^2^ = 0.073 (see Table 7), suggesting that the corrosion tests did not significantly influence the electrical resistance over time. In Table 8, the analysis indicated that there is no significant main effect of corrosion on electrical resistance values, F(1, 14) = 1.103, *p* = 0.311, and eta^2^ = 0.073, implying that electrical resistance remained stable during the corrosion testing. The interaction effect between corrosion and sample type was non-significant, F(1, 14) = 0.031, *p* = 0.862, and eta^2^ = 0.002, showing no modification in electrical resistance between the two sample types due to corrosion testing. Therefore, from the MANOVA analysis, it can be concluded that both PV cell types differ in electrical resistance values, *p* > 0.05.

## 4. Discussion

**Figure 4:**
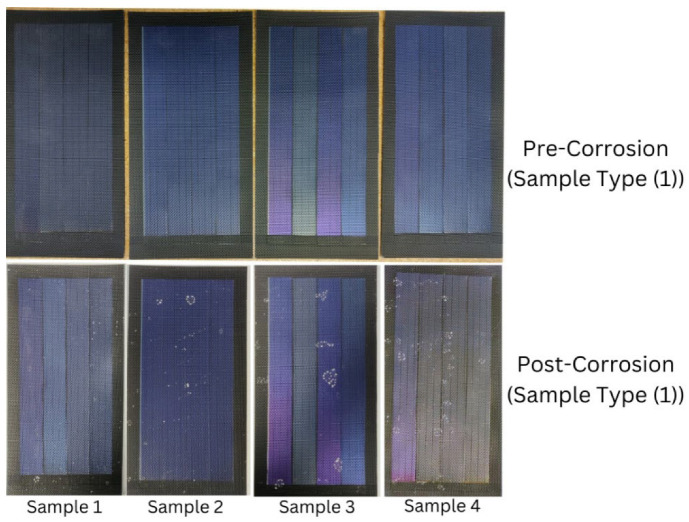
Conditions of four samples from sample type (1) pre- and post-corrosion, respectively.

**Figure 5:**
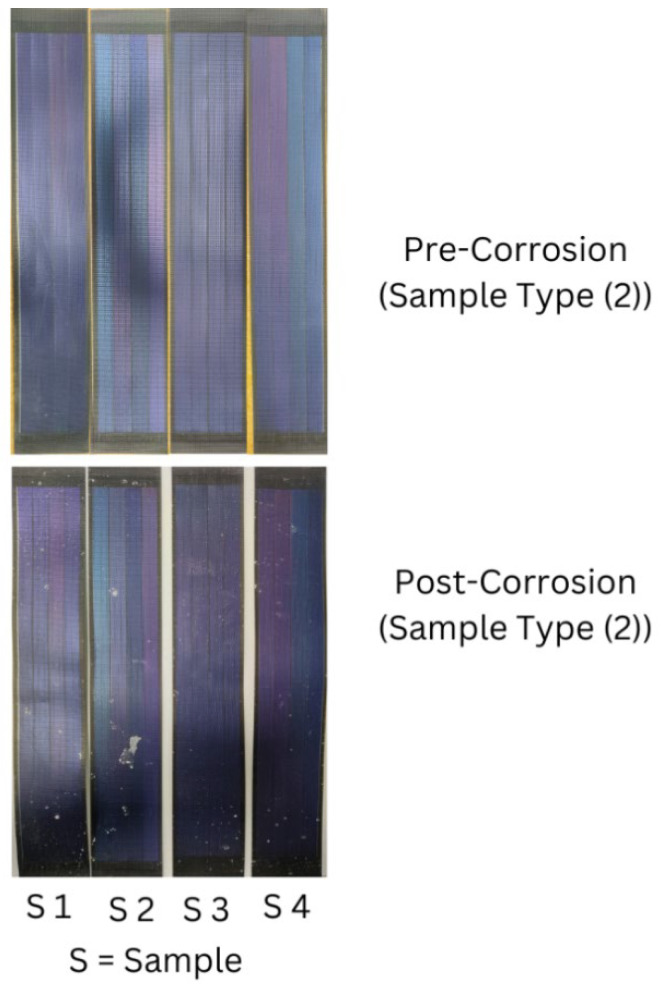
Conditions of four samples from sample type (2) pre- and post-corrosion, respectively.

This study compares commercially sourced wearable PV cells’ maximum current and electrical resistance pre- and post-corrosion testing conditions. A paired sample *t*-test assessed the current consistency and electrical resistance variations before and after corrosion testing. The researcher conducted a repeated MANOVA test to analyze the data and determine the comparative performance of the two sample types. This comparison will elucidate the relevance of durable, sustainable, and practical wearable PV cell-integrated textiles for the marine environment. The findings from the *t*-test indicated a significant difference in current and electrical resistance values, which means that the current values decreased, and the electrical resistance values increased significantly after corrosion for sample type (1). However, regarding sample type (2), the researcher concluded that corrosion testing had no significant effect on current and electrical resistance. Again, regarding current values, the findings from the MANOVA indicated an overall significant reduction in current readings across the corrosion testing after analyzing combined data from both sample types; however, the magnitude of reduction does not differ significantly between them. On the other hand, regarding electrical resistance values, the findings from the MANOVA indicated that the effect of corrosion testing on electrical resistance values did not differ significantly between the two sample types. In addition, corrosion testing did not substantially impact electrical resistance when both sample types were combined. The PV cell’s condition did not deteriorate from visual observations during corrosion, but salt is present due to salt spray during corrosion testing. These PV cells do not deteriorate, which might be because the materials of these PV cells are durable enough to withstand corrosion testing conditions (see Figure 4 and Figure 5).

### 4.1. Analysis of Current Value Variations in PV Samples Pre- and Post-Corrosion

The paired sample *t*-test showed a statistically significant difference between the pre- and post-current consistency values for sample type (1). This behavior was also observed in the prior literature, which showed that exposure to corrosive environments can lead to delamination within the PV layers. The delamination caused by corrosion compromises the integrity of the solar cell panel and can lead to reduced electrical conductivity and decreased light absorption, resulting in lower current output [20,23]. However, for sample type (2), pre- and post-current values had no significant difference. While searching for reasons, it was found that material composition could not be an issue as these samples are made from the same Ethylene Tetrafluoroethylene (ETFE) material. Regarding protective coatings or layers, manufacturers used a three-layer composite technology on these cells, which is also true for both types. The only reason could be their sizes or dimensions, as both samples’ dimensions and their surface areas are different. Due to the dimension, the resilience of type (2) may be more significant, which helps it to maintain better structural integrity under corrosion. Therefore, sample type (2) has a stronger resilience to corrosion, but type (1) has a low surface area, which makes type (2) potentially more suitable for long-term applications in corrosive marine environments.

After analyzing combined data from both sample types, the MANOVA test showed an overall significant reduction in current readings across the corrosion testing; however, the magnitude of reduction does not differ significantly between them. The minimal interaction between sample type and corrosion testing indicates that the materials and protective coatings employed in both PV cell designs exhibit similar vulnerability to corrosion, likely attributable to analogous material compositions or structural characteristics, as these coatings serve as barriers, obstructing corrosive agents from accessing the underlying materials and impeding corrosive reactions [35]. So, it can be concluded that protective coatings and corrosion-resistant materials are essential for mitigating the impact of corrosion and preserving the long-term performance of PV cell panels [20].

### 4.2. Analysis of Electrical Resistance Value Variations in PV Samples Pre- and Post-Corrosion

The findings from the *t*-test indicated a significant difference in electrical resistance values, which means those values increased significantly after corrosion for sample type (1). This behavior was also observed in the prior literature, which showed that exposure to corrosive environments can lead to increased electrical resistance, reducing current flow and decreasing overall PV cell performance [26]. In addition, this justifies decreasing current consistency values after corrosion testing. However, regarding sample type (2), the researcher concluded that corrosion testing had no significant effect on electrical resistance. As mentioned earlier, the reason could be their dimensions, as the dimensions of both samples are different. Due to the dimensions, the resilience of type (2) may be more significant, which helps it maintain better structural integrity under corrosion testing. Therefore, this justifies the stable current consistency values after corrosion testing for type (2).

Again, the findings from the MANOVA revealed no significant interaction between corrosion procedures and types. Corrosion testing did not substantially impact electrical resistance when both sample types were combined. The salt spray corrosion tests could not have lasted long enough to cause noticeable corrosion effects on either sample type, which might be one explanation for this behavior. Since the researcher was only there for a day, corrosion processes may take time to manifest and result in observable deterioration. As a result, a relatively short testing period could not have been enough to generate meaningful variations across the sample types. One main limitation of this research is the absence of standards in wearable electronics. The wearable electronics industry has been eager to develop devices with enhanced safety and performance attributes, yet the absence of reliable, standardized testing procedures may have hindered their ability to meet consumer expectations. Dependable standardization approaches for wearable electronics are crucial due to the inherent conflict between the two primary disciplines involved: textiles and electronics [36]. Another limitation of this research is that measuring current at various angles, such as 15°, 30°, and 45°, could yield more robust results. The sample sizes constrained the generalizability of the results. A greater diversity of samples could yield more robust conclusions. Future study directions may involve acquiring additional samples from various brands for comparative analysis to evaluate performance efficacy. In addition, in the future, advanced characterization techniques (e.g., SEM) could be used to study the corrosion effects at a microstructural level. A further disadvantage is that the researcher cannot regulate the laboratory’s environmental conditions, jeopardizing the test results. The researcher could not regulate the laboratory temperature during the corrosion test, which may have influenced the outcomes. Finally, the researcher gathered data solely for 24 h; it is advisable to extend this duration to 48, 72, and 108 h to ascertain the corrosion timeline of the PV cells, as advised for future investigations.

## 5. Conclusions

Wearable PV cells have been widely adopted as manufacturers can incorporate flexible, cost-effective, and lightweight PV cells onto clothing to harness and convert sunlight into electrical energy. These cells enable various uses, such as energy-harvesting garments, safety gear, protective fabrics, and military uniforms. However, most companies produce wearable PV for terrestrial applications, not marine ones, because applying these cells in marine environments is challenging due to the harsh, corrosive environment. Corrosion causes a reduction in current values in several PV cells for different applications, but the performance of flexible wearable PV cells for textiles remains uncertain. Conducting an electrical resistance test after a corrosion evaluation of PV cells is essential for determining the impact of corrosion on electrical efficiency and durability, as corrosion can elevate electrical resistance, hence diminishing current flow and total PV cell performance. This study compared commercially sourced wearable PV cells’ maximum current and electrical resistance pre- and post-corrosion testing conditions. A paired sample *t*-test assessed the current consistency and electrical resistance variations before and after corrosion testing. The researcher conducted a repeated MANOVA test to analyze the data and determine the comparative performance of the two sample types. The findings from the *t*-test indicated a significant difference in current and electrical resistance values, which means current values decreased and electrical resistance values increased significantly after corrosion for sample type (1) but not for sample type (2). Again, regarding current values, the findings from the MANOVA indicated an overall significant reduction in current readings across the corrosion testing after analyzing combined data from both sample types; however, the magnitude of reduction does not differ significantly between them. Regarding electrical resistance values, the findings from the MANOVA indicated that the effect of corrosion testing on electrical resistance values did not differ significantly between the two sample types. Finally, corrosion testing did not substantially impact electrical resistance when both sample types were combined. This comparison will elucidate the relevance of durable, sustainable, and practical wearable PV cells for textiles for the marine environment.

## Figures and Tables

**Table 1:** Mean and standard deviation values of current between two sample types in corrosion testing conditions.

Samples	Variables	M ^1^	SD ^1^
Sample Type (1)	Pre-current consistency (CC)	183.01	8.11
	Post-CC	181.31	8.12
Sample Type (2)	Pre-CC	271.25	7.60
	Post-CC	270.61	8.57

^1^ M = Mean; SD = Standard deviation.

**Table 2:** Significance levels of current values between the cycles of two sample types in corrosion testing conditions.

Samples	Variables	N ^1^	M ^1^	SD ^1^	Df ^1^	CI (2-Tailed, α = 0.05) ^1^	*p* ^1^	t ^1^
Sample Type (1)	Pre- and Post-CC	8	1.70	1.09	7	95%	0.003	4.41
Sample Type (2)	Pre- and Post-CC	8	0.64	1.31	7	95%	0.211	1.377

^1^ N = No. of samples; M = Reductions in mean values; SD = Standard deviation; Df = Degrees of freedom; CI = Confidence interval; *p* = *p*-value; t = t-static; CC = Current consistency.

**Table 3:** Mean and standard deviation values of electrical resistance readings between two sample types in corrosion testing conditions.

Samples	Variables	M ^1^	SD ^1^
Sample Type (1)	Pre-electrical resistance (ER)	0.313	0.099
	Post-ER	0.333	0.093
Sample Type (2)	Pre-ER	0.417	0.230
	Post-ER	0.430	0.224

^1^ M = Mean; SD = Standard deviation.

**Table 4:** Significance levels of electrical resistance values between the cycles of two sample types in corrosion testing conditions.

Samples	Variables	N ^1^	M ^1^	SD ^1^	Df ^1^	CI (2-Tailed, α = 0.05) ^1^	*p* ^1^	t ^1^
Sample Type (1)	Pre- and post-ER	8	0.020	0.014	7	95%	0.041	2.507
Sample Type (2)	Pre- and post-ER	8	0.013	0.080	7	95%	0.533	0.623

^1^ N = No. of samples; M = Reductions in mean values; SD = Standard deviation; Df = Degrees of freedom; CI = Confidence interval; *p* = *p*-value; t = t-static; CC = Current consistency.

**Table 5:** Current values pre- and post-corrosion across types.

Effect	Wilks’ Lambda Value	F ^1^	*p* ^1^	eta^2 1^
Corrosion	0.482	15.059	0.002	0.518
Corrosion × Types	0.818	3.111	0.100	0.182

^1^ F = F-statistic; *p* = *p*-value; eta^2^ = partial eta squared.

**Table 6:** Significance in current values across corrosion.

	SS ^1^	Df ^1^	MS ^1^	F ^1^	*p* ^1^	eta^2 1^
Corrosion	10.928	1	10.928	15.059	0.002	0.518
Corrosion × Types	2.258	1	2.258	3.111	0.100	0.182
Error (Corrosion)	10.159	14	0.726			

^1^ SS = Type III Sum of Squares; Df = degrees of freedom; MS = Mean Square; F = F-statistic; *p* = *p*-value; eta^2^ = partial eta squared.

**Table 7:** Effects in electrical resistance values pre- and post-corrosion for both types.

Effect	Wilks’ Lambda Value	F ^1^	*p* ^1^	eta^2 1^
Corrosion	0.927	1.103	0.311	0.073
Corrosion × Types	0.998	0.031	0.862	0.002

^1^ F = F-statistic; *p* = *p*-value; eta^2^ = partial eta squared.

**Table 8:** Significance in electrical resistance across corrosion testing.

	SS ^1^	Df ^1^	MS ^1^	F ^1^	*p* ^1^	eta^2 1^
Corrosion	0.002	1	0.002	1.103	0.311	0.073
Corrosion × Types	0.000053	1	0.000053	0.031	0.862	0.002
Error (Corrosion)	0.023	14	0.002			

^1^ SS = Type III Sum of Squares; Df = degrees of freedom; MS = Mean Square; F = F-statistic; *p* = *p*-value; eta^2^ = partial eta squared.

## Data Availability

The original contributions presented in this study are included in the article. Further inquiries can be directed to the corresponding author.
